# Absent in melanoma 1-like (AIM1L) serves as a novel candidate for overall survival in hepatocellular carcinoma

**DOI:** 10.1080/21655979.2021.1939636

**Published:** 2021-06-15

**Authors:** Wenliang Zhou, Yuan Zhang, Shixi Zhang, Zongguo Yang

**Affiliations:** a Department of Infectious Diseases, Shangqiu Municipal Hospital, Shangqiu, He’nan, China; b Department of Integrative Medicine, Shanghai Public Health Clinical Center, Fudan University, Shanghai, China

**Keywords:** AIM1L, hepatocellular carcinoma, overall survival, absent in melanoma 1-like

## Abstract

Identifying biomarkers for hepatocellular carcinoma (HCC) survival is of great importance for the early detection, monitoring, and predicting for prognosis. This study aimed to investigate the candidate biomarkers for predicting overall survival (OS) in HCC patients. Using RTCGAToolbox, top 50 upregulated differential expressed genes (DEGs) were identified. The least absolute shrinkage and selection operator (LASSO) and Cox models were used to select powerful candidate genes, and log rank method was used to address the survivor functions of potential biomarkers. Selected by LASSO model, ANLN, TTK, AIM1L and person neoplasm cancer status might be candidate parameters associated with OS in HCC patients. After adjusting person neoplasm cancer status, ANLN and TTK levels in Cox model, AIM1L was identified as a risk factor for predicting OS in HCC patients (HR = 1.5, *P* = 0.037). Validated in The Cancer Genome Atlas (TCGA), International Cancer Genome Consortium (ICGC) and Gene Expression Omnibus (GEO) series, AIM1L was significantly overexpressed in tumor tissues compared to nontumor tissues (all *P* < 0.0001). HCC patients with high AIM1L in tumor tissues had significantly unfavorable OS compared to those with low AIM1L in TCGA, ICGC, Gene Expression Profiling Interactive Analysis (GEPIA) and Kaplan-Meier Plotter datasets (all *P* < 0.05). Conclusively, AIM1L is upregulated in tumor samples and serves as a novel candidate for predicting unfavorable OS in HCC patients.

## Graphical abstract

**Figure:**
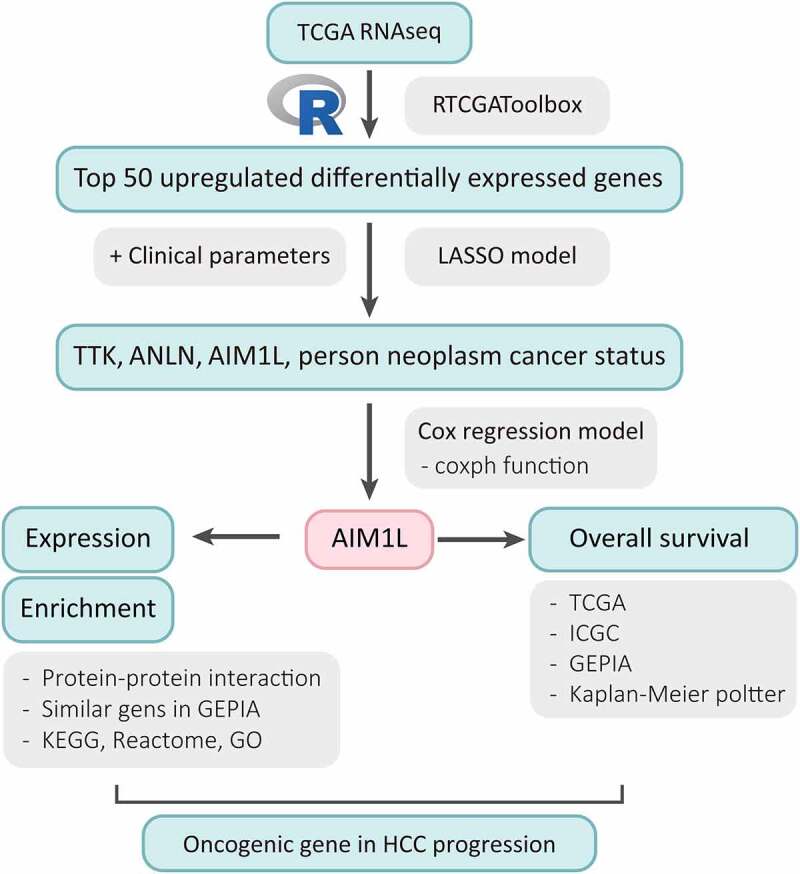

## 1 Introduction

Hepatocellular carcinoma (HCC) is one of the most frequently occurring human malignancies and the second leading cause of cancer-related deaths [1]. During the past few decades, the incidence of HCC continued to increase, and it will be estimated to rise over the next 10 to 20 years [2–4]. Even recent advances in the first-line systemic treatment of showed effective results for HCC patients, the overall prognosis of this population is still dull [5–7]. The liver cancers including HCC related mortality has marked increased by more than 2% annually since 2007 [2]. To identify potential biomarkers for HCC survival should be of great importance for the early detection, monitoring, and predicting for prognosis, which is also helpful for the understanding the pathological characteristics in HCC patients.

Several genetic candidates have been used in HCC surveillance, diagnosis, prognosis, and treatment responses [8–10]. With the development of high-throughput technologies and gene chips, and next-generation sequencing, the genomic pattern of HCC has been determined which greatly improved our understanding of genetic and epigenetic changes and their interaction in the HCC aggressiveness [8,11–14]. All these approaches have become fast approaches to identify differentially expressed genes (DEGs) and functional pathways, and led to a dramatic increase in the accessibility of molecular insights at multiple biological levels involved in the HCC development [13,15–18]. Recently, these bioinformatics data repositories have rapidly evolved into an essential aid for molecular hepatology [15]. The availability of genome sequencing data from liver tumors provides us with valuable resources, which is vital to help us to facilitate the identification of promising biomarkers or therapeutic targets for HCC population [14].

In our study, the top 50 upregulated DEGs between tumor and nontumor samples in HCC patients were identified using RTCGAToolbox package in The Cancer Genome Atlas (TCGA) dataset [19]. The least absolute shrinkage and selection operator (LASSO) model and Cox proportional hazards regression model were used to investigate and validate the potential candidates [20]. The aim of this study is to screen promising candidates for predicting overall survival (OS) in HCC patients with integrative bioinformatic approaches.

## 2 Materials and methods

### 2.1 Data source

Using the ‘getFirehoseData’ function in the RTCGAToolbox package [19], gene expression data calculated by RNAseq from HCC patients were downloaded from the Firehose project when dataset was set as ‘LIHC’, runDate was set as ‘20,160,128’, and RNAseqGene means ‘TRUE’. To assess the DEGs between the normal and tumor samples in HCC patients in the TCGA dataset, the ‘getDiffExpressedGenes’ function was addressed with criteria *P* value < 0.05, adjusted *P* value < 0.05 and logFC ≥ 2. Top 50 upregulated DEGs was obtained for heatmap performance when ‘hmTopUpN’ equals to 50 and ‘hmTopDownN’ equals to 0.

### 2.2 Patients

The clinical and gene expression data with Z scores of Liver Hepatocellular Carcinoma (LIHC, TCGA, PanCancer Atlas) dataset was obtained from cBioPortal for cancer genomics [21,22]. After restricting tumor pathological type as HCC and matching gene expression levels with clinical data, 366 HCC patients were included in this study. Clinico-pathological characteristics including age, gender, American Joint Committee on Cancer (AJCC) staging, new tumor even after initial treatment, pathological TNM stages, person neoplasm cancer status, race, radiation therapy, and weight were available. Every participant provided verified informed consent, as declared in the original dataset.

In the International Cancer Genome Consortium (ICGC, https://daco.icgc.org/) database, liver cancer project LIRI-JP with HCC subtype was included in this study [23]. 260 HCC donors were available. After matching the gene expression data and survival information, and removing subjects with OS less than 20 days, 235 cases were included in the final analysis.

### 2.3 Gene expression analysis

Raw.CEL files of the microarray datasets in Gene Expression Omnibus (GEO, https://www.ncbi.nlm.nih.gov/geo/) were downloaded and normalized by quantile method of Robust Multichip Analysis from the R affy package [24]. k-Nearest Neighbor method by impute function was used to impute the missing gene expression data [25]. Gene expression levels between the normal and tumor samples were calculated by the Limma package [26].

In the TCGA dataset, mRNA normalized counts data of LIHC derived from RNAseq Htseq platform were downloaded from Genomic Data Commons Data Portal (https://cancergenome.nih.gov/). The edgeR package in R program were used to identify gene expression levels between tumor and nontumor tissues [27,28].

In the ICGC dataset, mRNA normalized counts data of 237 tumor samples and 197 normal liver samples in LIRI-JP project, which was derived from Illumina HiSeq platform, were downloaded from ICGC data portal (https://dcc.icgc.org/)[23].

### 2.4 LASSO model establishment

LASSO regression model was used to determine the most powerful prognostic markers for OS in HCC patients [20]. In the TCGA dataset, parameters including age, gender, tumor status, AJCC staging, weight, radiation therapy, race, pathological status, and top 50 upregulated DEGs were included in the LASSO model. ‘glmnet’ and ‘survival’ packages were used for LASSO model establishment with family equals to ‘cox’ and alpha equals to 1. The model was validated with 5-fold cross-validation. Both ‘lambda.1se’ and ‘lambda.min’ were used to assess the coefficient of parameters [29].

### 2.5 Functional enrichment of candidate genes

Protein-protein interaction (PPI) analysis of the candidate genes were addressed in STRING [30] and STITCH databases [31]. Top 100 similar genes of the candidate genes were respectively searched from LIHC tumor, LIHC normal and Genotype-Tissue Expression (GTEx) liver datasets in GEPIA database [32]. All these AIM1L-related genes were screened by edgeR package with |log FC| > 1, and adjusted *P* value < 0.05 [27,28], differentially expressed genes were enrolled in the Gene Set Enrichment Analysis (GSEA) [33]. Investigate gene sets module in the Molecular Signatures Database (MSigDB) v7.4 in GSEA were used for Kyoto Encyclopedia of Genes and Genomes (KEGG) pathway, Reactome, and Gene Ontology (GO) enrichment [34,35]. Top ten genesets were included with a false discovery rate (FDR) q-value less than 0.05.

### 2.6 Statistical analysis

Differences of gene levels between the individual groups were analyzed using student *t* test or Mann–Whitney *U* test based on data types. Parameters enrolled in LASSO model were included in Cox proportional hazards regression model. Results were reported as hazard ratios (HR) with 95% confidence interval (95% CI). Log rank method was used to address the survivor functions of candidate genes for OS in HCC patients. The Kaplan Meier plotter [36] and Gene Expression Profiling Interactive Analysis (GEPIA) [32] online services were also used to confirm the survival relationship. Stata software version 16.0 (STATA Corp., Texas, USA) was used. A two-tailed *P* < 0.05 were considered significance for all tests.

## 3 Results

The LASSO model showed that TTK, ANLN, AIM1L and person neoplasm cancer status should be underlying candidates of OS in HCC. Using integrated bioinformatic methods, we found that AIM1L was at a low level in normal livers and significantly overexpressed in HCC tumor tissues. Cox regression model and Kaplan-Meier analysis in multiple datasets indicated that high levels of AIM1L in tumors contributed to unfavorable OS in HCC patients. Functional enrichment revealed that AIM1L-related genes might involve in cell proliferation and cell migration. We assumed that AIM1L exerts oncogenic roles in HCC progression.

### 3.1 Top 50 upregulated candidate genes identified by RTCGAToolbox

**Figure 1.**
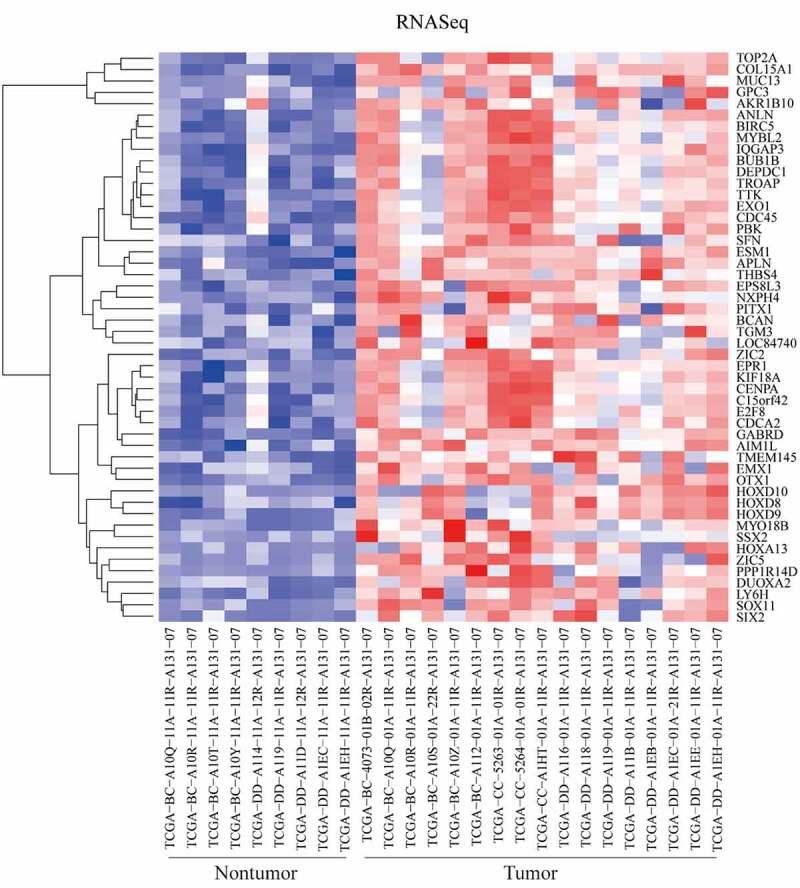
Differentially expressed genes (DEGs) between tumor and nontumor tissues in HCC patients in TCGA dataset were screened the ‘getDiffExpressedGenes’ function in RTCGAToolbox package with criteria *P* value < 0.05, adjusted *P* value < 0.05 and logFC ≥ 2. Top 50 upregulated DEGs was obtained for heatmap performance when ‘hmTopUpN’ equals to 50 and ‘hmTopDownN’ equals to 0

Detected by the RTCGAToolbox package in R program, top 50 upregulated DEGs in tumor tissues were identified. The heatmap of these DEGs between tumor and nontumor samples were presented in Figure 1.

### 3.2 Selection of powerful biomarkers for OS in HCC patients by LASSO and Cox

**Figure 2.**
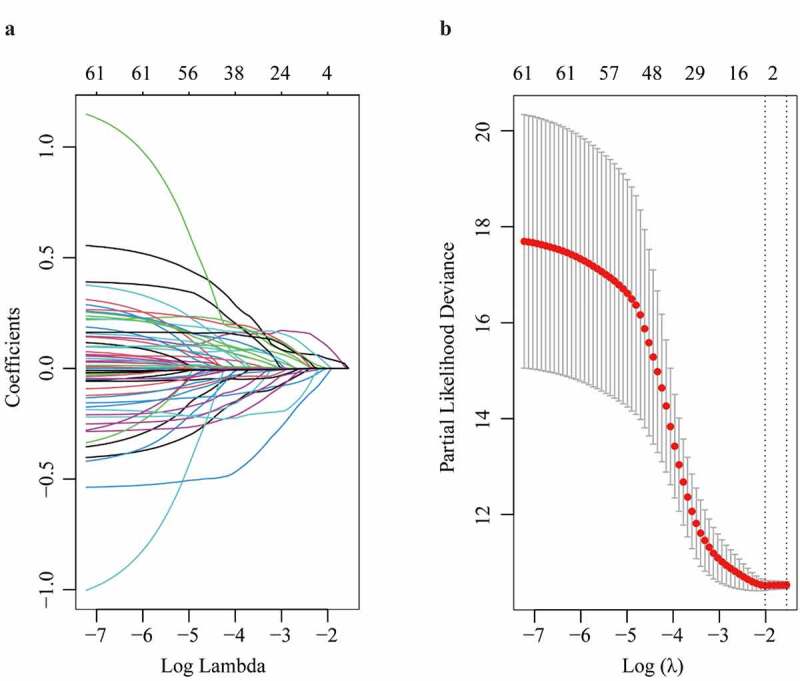
Parameter selection through LASSO regression (a) and elucidation of LASSO coefficient profiles for selected factors (b)

In the TCGA dataset, the top 50 upregulated DEGs, together with clinico-pathological characteristics including age, gender, AJCC staging, new tumor even after initial treatment, pathological TNM stages, person neoplasm cancer status, race, radiation therapy, and weight were enrolled in LASSO model (Figure 2a). After the 5-fold cross validation, parameters including TTK, ANLN, AIM1L and person neoplasm cancer status were recruited to be underlying candidates of OS in HCC patients when λ took the minimum value (Figure 2a). The regression coefficient plot of factors by LASSO was shown in Figure 2b.

**Figure 3.**
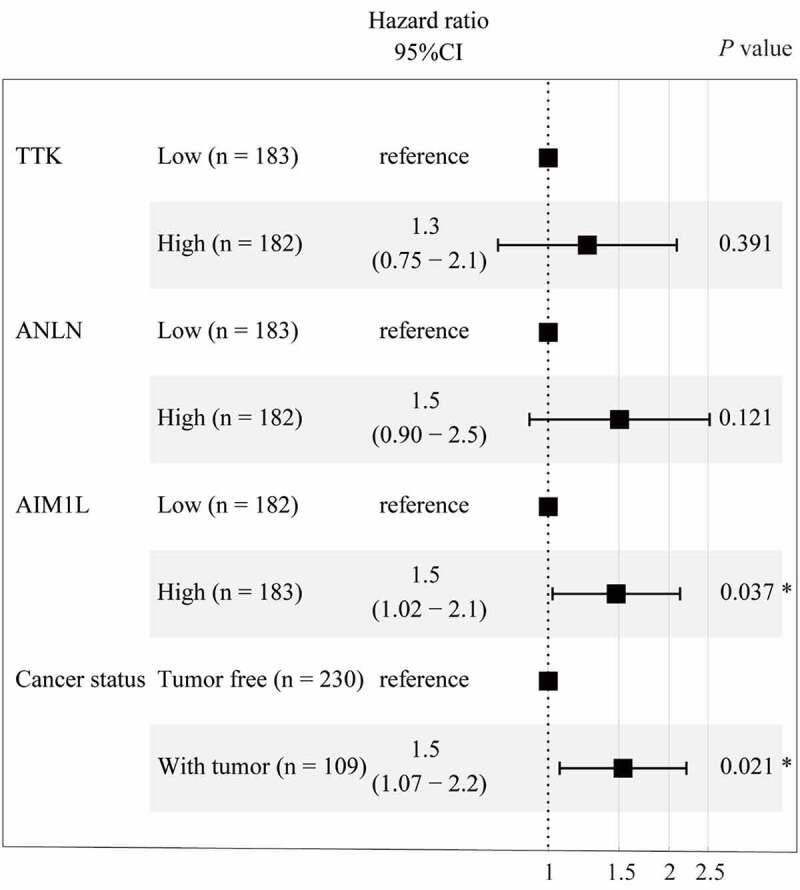
Cox proportional hazards regression model using the ‘coxph’ function in R program was established. Four parameters including TTK, ANLN, AIM1L, and cancer status screened by LASSO model were included in Cox regression model for OS in HCC patients. After adjusting TTK and ANLN, AIM1L and cancer status were significantly associated with OS in HCC patients (both *P* < 0.05)

To fit a Cox proportional hazards regression model, the ‘coxph’ function in R program was used. As summarized in Figure 3, AIM1L serves as an independent prognosis predictor for OS in HCC patients after adjusting TTK, ANLN and person neoplasm cancer status (HR = 1.5, 95%CI = 1.02–2.1, *P* = 0.037, Figure 3).

### 3.3 AIM1L expression

**Figure 4.**
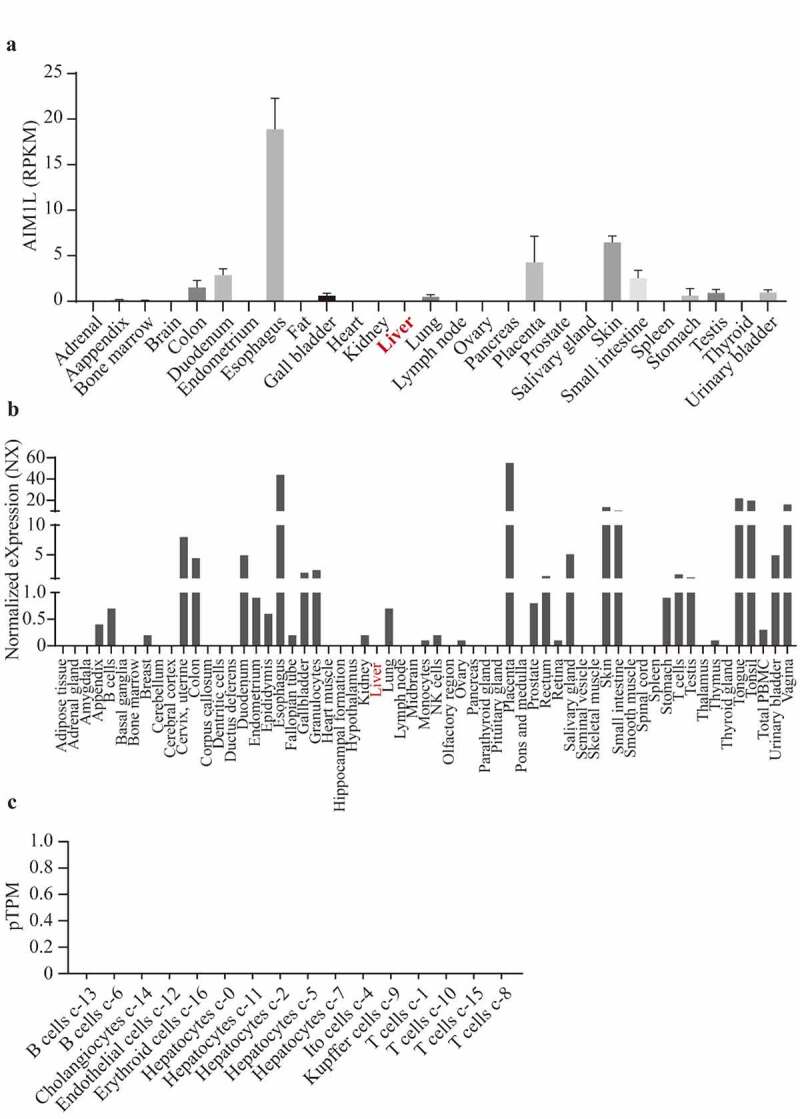
Through searching AIM1L in PubMed (gene ID: 55057), the gene expression levels in 27 normal tissues from Fagerberg’s dataset were obtained. In Consensus dataset in GEPIA database, normalized expression (NX) of AIM1L from three transcriptomics datasets, namely, Human Protein Atlas (HPA), the Genotype-Tissue Expression (GTEx) project and the Functional Annotation of Mammalian Genomes 5 (FANTOM5) project, was calculated. AIM1L is at low levels in normal liver both in Fagerberg’s dataset (a) and Consensus dataset from HPA (b). In HPA database, single cell RNA sequencing indicated that AIM1L was not detected in Hepatocytes, Cholangiocytes, Endothelial cells, Erythroid cells, Ito cells, Kupffer cells, T cells and B cells in liver tissue (c)

In Fagerberg’s report, RNAseq was performed to determine tissue-specificity of protein-coding genes of tissue samples from 95 human individuals representing 27 different tissues [37]. In Consensus dataset, normalized expression (NX) of the candidate from three transcriptomics datasets, namely, Human Protein Atlas (HPA), the Genotype-Tissue Expression (GTEx) project and the Functional Annotation of Mammalian Genomes 5 (FANTOM5) project, was calculated. As shown in Figure 4, AIM1L was expressed at low levels in normal liver tissues in Fagerberg’s dataset and Consensus dataset (Figure 4a and b). In addition, single cell RNA sequencing indicated that AIM1L was not detected in Hepatocytes, Cholangiocytes, Endothelial cells, Erythroid cells, Ito cells, Kupffer cells, T cells and B cells in liver tissue (Figure 4c).

**Figure 5.**
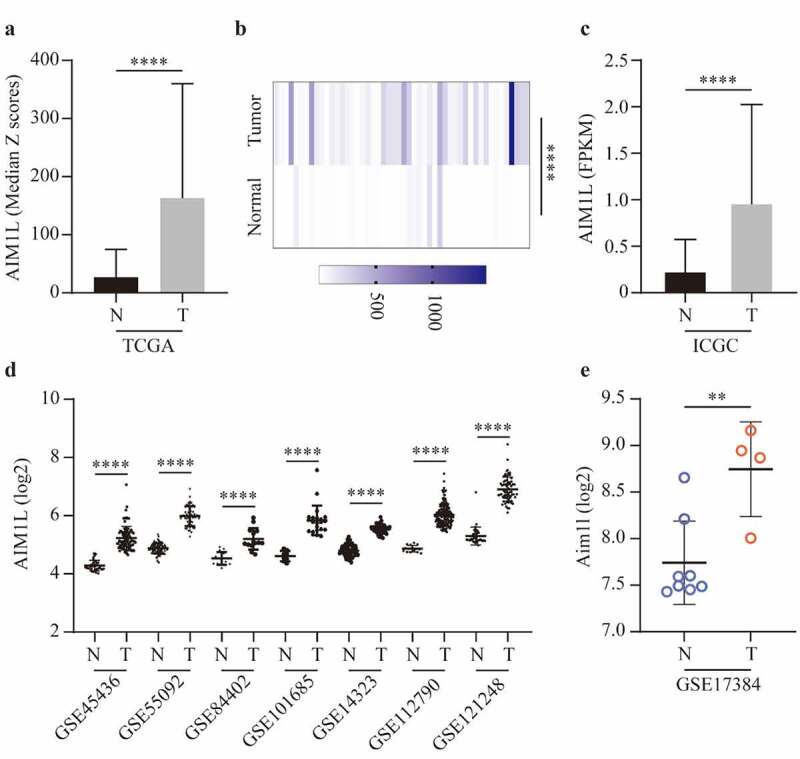
AIM1L expression between tumor and nontumor samples in HCC patients were compared. AIM1L was significantly upregulated tumor tissues in TCGA (*P* < 0.0001, A), in 50 paired tumor samples from TCGA (*P* < 0.0001, B), in LIRI-JP project from ICGC (*P* < 0.0001, C) and in GEO series including GSE45436, GSE55092, GSE84402, GSE101685, GSE14323, GSE112790, and GSE121248 (all *P* < 0.0001, D). In a rat model naturally occurring hepatotumorigenesis induced by oxidative stress, AIM1L was significantly upregulated in liver cancer tissues compared to normal liver tissues (*P* < 0.01, E)

Next, we compared the AIM1L expression between tumor and nontumor samples. In the TCGA dataset, AIM1L mRNA was significantly upregulated in tumor tissues compared to nontumor tissues (*P* < 0.0001, Figure 5a). In the 50 paired normal and tumor samples extracted from TCGA dataset, AIM1L was also significantly overexpressed in tumor samples compared to normal livers (*P* < 0.0001, Figure 5b). Additionally, AIM1L mRNA was significantly upregulated in tumor tissues compared to nontumor tissues in LIRI-JP project from ICGC (*P* < 0.0001, Figure 5c) and GEO series including GSE45436 [38], GSE55092 [39], GSE84402 [40], GSE101685 [41], GSE14323 [42], GSE112790 [43], and GSE121248 [44] (all *P* < 0.0001, Figure 5d). In a rat model naturally occurring hepatotumorigenesis induced by oxidative stress [45], AIM1L was significantly upregulated in liver cancer tissues compared to normal liver tissues (*P* < 0.01, Figure 5e).

### 3.4 High AIM1L accounts for unfavorable OS in HCC patients

**Figure 6.**
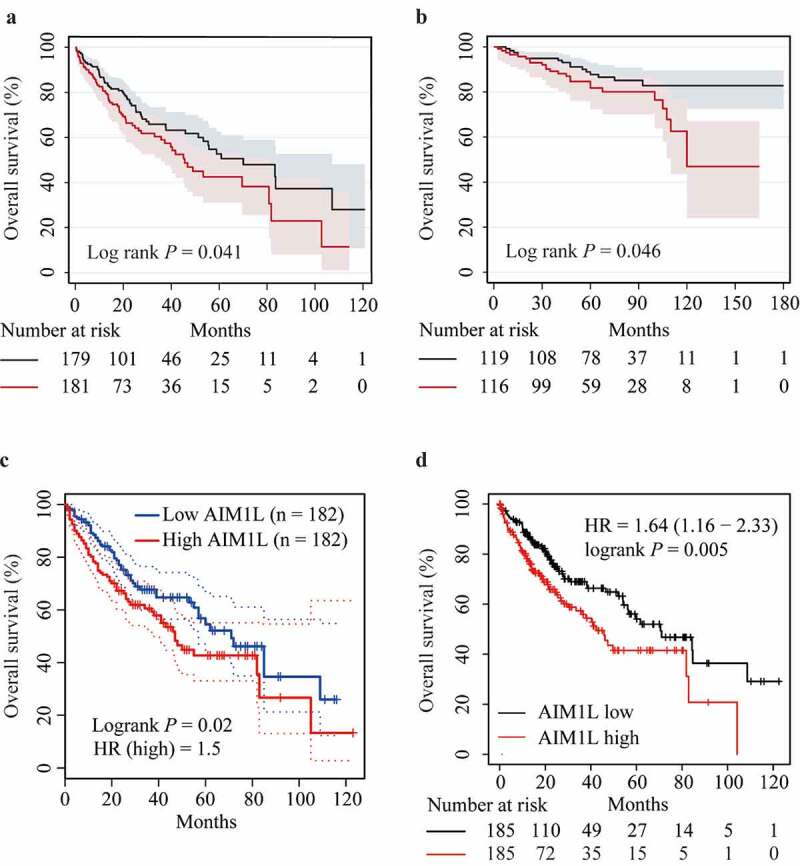
Associations between AIM1L and HCC survival. Kapan-Meier plot method indicated that HCC patients with AIM1L overexpression in tumor tissues had unfavorable OS compared to those with low levels of AIM1L in TCGA dataset (Log rank *P* = 0.041, A), ICGC dataset (Log rank *P* = 0.046, B), GEPIA (HR = 1.5, Log rank *P* = 0.02, C) and Kapan-Meier plotter (HR = 1.64, 95% CI = 1.16–2.33, Log rank *P* = 0.041, D) datasets

As described in Figure 6, HCC patients from the TCGA dataset with high AIM1L in tumor tissues had significantly unfavorable OS compared to those with low AIM1L (*P* = 0.041, Figure 6a). In the LIRI-JP project from the ICGC database, HCC patients with high AIM1L in tumor tissues had significantly poorer OS than those with low AIM1L (*P* = 0.046, Figure 6b).

In the GEPIA database, high AIM1L might contribute to significantly worse OS in HCC patients (HR = 1.5, *P* = 0.02, Figure 6c). Similarly, HCC patients with high AIM1L levels in tumor tissues had higher risk for unfavorable OS compared to those with low AIM1L in the Kaplan-Meier Plotter database (HR = 1.64, 95% CI = 1.16–2.33, *P* = 0.005, Figure 6d). Considered results from Cox model and Kaplan-Meier analysis, we assumed that AIM1L serves as a novel biomarker for predicting unfavorable OS in HCC patients.

### 3.5 Functional enrichment of AIM1L-related genes

**Figure 7.**
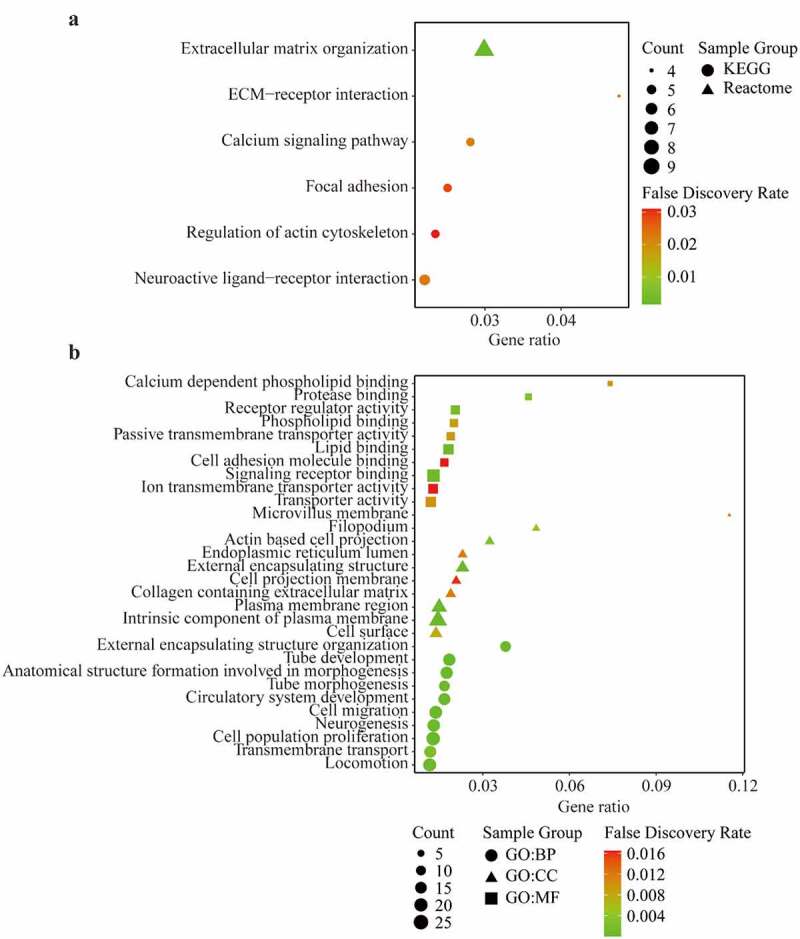
KEGG pathway, Reactome (a) and Gene Ontology (GO) enrichment (b) including Biological process (BP), Cellular component (CC), and Molecular function (MF) of differentially expressed related-genes of AIM1L screened by edgeR package in TCGA dataset with a |log FC| > 1, and adjusted *P* value < 0.05. Top ten genesets were addressed with a false discovery rate (FDR) q-value < 0.05

The PPI of AIM1L was presented in Supplementary Figure S1. The similar genes of AIM1L in LIHC tumor, LIHC normal and GTEx datasets in GEPIA platform were summarized in Supplementary Table S1. Whether these AIM1L-related genes were differentially expressed between tumor and nontumor tissues were screened using the edgeR package in R program. Totally, 144 AIM1L-related DEGs were selected in the functional enrichment analysis (Supplementary Figure S2). As described in Figure 7, AIM1L-related genes involved in the process of extracellular matrix (ECM) organization, ECM-receptor interaction, calcium signaling pathway, focal adhesion, regulation of action cytoskeleton, and neuroactive ligand-receptor interaction (all FDR < 0.05, Figure 7a). GO enrichment revealed that AIM1L-related genes implicated in multiple biological processes (BP) including cell proliferation and cell migration (Figure 7b). The GO cellular component (CC), and molecular function (MF) of AIM1L-related genes were also summarized in Figure 7b (all FDR < 0.05).

## 4 Discussion

Previously, AIM1L mRNA overexpression was identified in several cancer cell lines including prostate cancer, bladder inverted papilloma, bladder cancer, colon cancer, pancreas cancer, ovarian cancer, endometrial cancer, and breast cancer. While only placenta and testis exhibited high AIM1L mRNA expression among noncancerous tissues [46]. A pharmacogenomics study indicated that the risk of nausea and vomiting in opioid-treated cancer patients has a genetic component. Whole exome sequencing of DNA pools revealed that six single nucleotide polymorphisms in some genes including AIM1L were associated with nausea and vomiting in opioid-treated cancer patients [47]. Unfortunately, impact of AIM1L on tumorigenesis is rarely investigated. Hence, the roles of AIM1L in the human cancers including HCC population are still essentially needed to be elucidated.

AIM1L mRNA was not detected in normal liver tissues [46], which is consistent with our results. AIM1L mRNA was significantly upregulated in tumor samples according to the findings from our bioinformatic study enrolled several public datasets. In addition, high AIM1L mRNA levels were significantly correlated with OS in HCC patients, both in TCGA and ICGC databases. Our functional enrichment of AIM1L-related genes indicated that AIM1L and its-related genes might involve in cell proliferation, cell migration, and signaling pathways, namely, extracellular matrix organization, ECM-receptor interaction, calcium signaling pathway, etc. Since tumorigenesis roles of AIM1L in human cancers have not been reported in details, absent in melanoma 1 (AIM1) has been studies in various human malignancies [48–50]. AIM1 has been shown to be highly overexpressed in prostate cancer tissues, and cultured androgen-independent prostate cancer cells, indicating that AIM1 might be a potential therapeutic target for treatment of prostate cancer [51]. Among 93 bladder cancers samples and 26 nonmalignant tissues, the frequencies of AIM1 methylation were significantly higher in tumors (84%) than that in nontumor tissues (27%) [52]. The AIM1 methylation was also shown to be correlated with nasopharyngeal carcinoma compared to controls [53]. And AIM1 methylation was significantly associated with invasive tumors [52]. Metastatic melanoma had higher frequency of AIM1 promoter hypermethylation than primary melanomas. Melanomas AIM1 methylation was correlated with disease-free survival (DFS) and OS in Stage I/II patients. Circulating methylated AIM1 was also detected in melanoma patients’ serum and was predictive of OS in Stage IV patients [49]. On the other hand, advanced prostate cancers usually own AIM1 deletion and reduced expression. AIM1 depletion in prostate epithelial cells increases cell migration and invasion, and anchorage-independent growth. AIM1 could also inhibit pro-invasive properties in benign prostate epithelium. Moreover, AIM1 downregulation results in high risk of metastatic dissemination in vivo [48]. AIM1 has also been identified as a potential suppressor candidate of human malignant melanoma [50]. Considered controversial results of AIM1 in human cancers and little literatures of AIM1L available, we assumed that further research focusing AIM1L on tumorigenesis in cancers including HCC should be urgently conducted.

This bioinformatic study has some limitations. First, no experiments were performed to address the effects of AM1L on hepatoma cellular functions. Second, no our own follow-up data of HCC patients were available, the predictive values of AIM1L for OS in HCC patients were not validated in prospective cohorts. Third, this analysis was conducted at mRNA level, links between AIM1L protein and HCC prognosis was not investigated.

## 5 Conclusion

AIM1L mRNA is at a low level in normal liver tissues, and is upregulated in tumor samples. High levels of AIM1L in tumors contributed to unfavorable OS in HCC patients. The AIM1L-related genes might involve in cell proliferation and cell migration. based on our results, we cautiously draw a hypothesis that AIM1L is an oncogenic gene and promotes cancer progression in HCC patients. Considered few reports of AIM1L on HCC tumor pathological phenotypes, we suggest that experimental and clinical research of the impact of AIM1L on the development of HCC should be addressed in future.

## Supplementary Material

Supplemental Material

## Data Availability

Datasets of the current study are available from the NCBI Gene Expression Omnibus (https://www.ncbi.nlm.nih.gov/geo/), The Cancer Genome Atlas (https://portal.gdc.cancer.gov/), and the International Cancer Genome Consortium (https://daco.icgc.org/) databases. All the datasets were available from the corresponding authors with reasonable request.

## Highlights

- AIM1L is low in normal liver and high in liver tumors.
- High AIM1L in tumors accounts for unfavorable OS in HCC patients.
- Enrichment analysis indicated that AIM1L might involve in cell proliferation and cell migration.
