# Supplementary material for: Absent in melanoma 1-like (AIM1L) serves as a novel candidate for overall survival in hepatocellular carcinoma
Source: Bioengineered. 2021 Jun 15;12(1):2750–62. doi: 10.1080/21655979.2021.1939636 (PMC8806546; doi:10.1080/21655979.2021.1939636)

**Absent in melanoma 1-like (AIM1L) serves as a novel candidate for overall survival in hepatocellular carcinoma**

**Supplementary Materials**

**Table S1** Similar genes of AIM1L in LIHC tumor, LIHC normal and GTEx datasets in GEPIA platform

| LIHC tumor | |  | LIHC normal | |  | GTEx | |
| --- | --- | --- | --- | --- | --- | --- | --- |
| Gene | PCC |  | Gene | PCC |  | Gene | PCC |
| PCBP4 | 0.47 |  | WI2-2610K16.2 | 0.93 |  | PDE10A | 0.91 |
| HDAC11 | 0.47 |  | Metazoa_SRP | 0.92 |  | RP11-54A9.1 | 0.9 |
| PPP1R35 | 0.45 |  | TPM1 | 0.92 |  | LIMS3L | 0.9 |
| SAMD10 | 0.44 |  | PDZK1IP1 | 0.92 |  | CREB5 | 0.89 |
| MIF | 0.42 |  | LINC00305 | 0.9 |  | LRRC32 | 0.89 |
| RP11-569G9.7 | 0.41 |  | SEMA7A | 0.89 |  | SLCO2A1 | 0.89 |
| AC133528.2 | 0.41 |  | SMPDL3B | 0.89 |  | HOXD1 | 0.89 |
| RP5-908M14.5 | 0.4 |  | TAC1 | 0.89 |  | RP11-1C1.6 | 0.88 |
| YDJC | 0.4 |  | TRNP1 | 0.89 |  | DPEP1 | 0.88 |
| AP5Z1 | 0.4 |  | KRT86 | 0.89 |  | KB-1517D11.4 | 0.88 |
| DDX51 | 0.4 |  | MMP24 | 0.88 |  | GABRD | 0.88 |
| RP11-496I9.1 | 0.4 |  | DTNA | 0.88 |  | CILP2 | 0.88 |
| RP11-120K24.5 | 0.39 |  | FSTL3 | 0.88 |  | PODXL | 0.88 |
| GDPD3 | 0.39 |  | RP11-205M5.3 | 0.87 |  | GREM1 | 0.87 |
| LYPLA2 | 0.39 |  | ADRA1D | 0.87 |  | SCG2 | 0.87 |
| EMG1 | 0.39 |  | BDKRB2 | 0.87 |  | COL11A1 | 0.87 |
| TOM1 | 0.38 |  | CAPN6 | 0.86 |  | STC2 | 0.87 |
| TOP1MT | 0.38 |  | LOXL4 | 0.86 |  | NOD1 | 0.87 |
| ZMAT5 | 0.38 |  | TSPAN15 | 0.86 |  | CHMP1B2P | 0.87 |
| KB-1572G7.2 | 0.38 |  | GPSM1 | 0.86 |  | MMP11 | 0.86 |
| EGFR-AS1 | 0.38 |  | DBNDD1 | 0.85 |  | RP4-809F18.1 | 0.86 |
| KRT23 | 0.38 |  | ST14 | 0.85 |  | LINC01436 | 0.86 |
| PUS1 | 0.38 |  | HSPB8 | 0.85 |  | FSTL3 | 0.86 |
| NAT9 | 0.38 |  | GATS | 0.85 |  | KRT86 | 0.86 |
| FGFR4 | 0.37 |  | APOBEC3B | 0.85 |  | RP11-12A20.7 | 0.86 |
| RP11-728F11.3 | 0.37 |  | NKX2-8 | 0.85 |  | CITF22-49D8.1 | 0.86 |
| REXO4 | 0.37 |  | PDGFA | 0.84 |  | LAMA4 | 0.86 |
| NHP2L1 | 0.37 |  | PAGE4 | 0.84 |  | RP11-1C1.4 | 0.86 |
| ITPA | 0.37 |  | SGK223 | 0.83 |  | RP11-443C10.1 | 0.85 |
| SLC35C2 | 0.37 |  | ANXA2 | 0.83 |  | SH3RF3-AS1 | 0.85 |
| PLA2G4F | 0.37 |  | TMEM171 | 0.83 |  | CHSY1 | 0.85 |
| STX3 | 0.37 |  | FAP | 0.83 |  | CRLF1 | 0.85 |
| KRT18 | 0.37 |  | GUCA2A | 0.83 |  | STMN3 | 0.85 |
| MSH5-SAPCD1 | 0.37 |  | FA2H | 0.83 |  | OSBPL3 | 0.85 |
| PRR7 | 0.36 |  | TTC9 | 0.83 |  | LZTS1 | 0.85 |
| AP006285.2 | 0.36 |  | PLEKHN1 | 0.83 |  | MTHFD2 | 0.85 |
| PXN-AS1 | 0.36 |  | VSIG2 | 0.83 |  | CTD-3035K23.7 | 0.85 |
| SPIRE2 | 0.36 |  | FAT1 | 0.82 |  | PTP4A3 | 0.85 |
| C6orf1 | 0.36 |  | RN7SL428P | 0.82 |  | RP11-244F12.3 | 0.85 |
| NPM3 | 0.36 |  | Metazoa_SRP | 0.82 |  | SLC25A36 | 0.85 |
| TRMT112 | 0.36 |  | RN7SL331P | 0.82 |  | KCNS2 | 0.84 |
| KRT8 | 0.36 |  | RAB11FIP1 | 0.82 |  | DOK5 | 0.84 |
| PSG4 | 0.36 |  | PLP2 | 0.82 |  | PDLIM4 | 0.84 |
| PQLC2 | 0.36 |  | TRBV24-1 | 0.82 |  | LYPD5 | 0.84 |
| PRPF6 | 0.36 |  | TUBB8P12 | 0.82 |  | CD109 | 0.84 |
| FOXJ1 | 0.36 |  | HYAL4 | 0.82 |  | STAC2 | 0.84 |
| RP1-90G24.11 | 0.36 |  | AC017104.2 | 0.81 |  | STC1 | 0.84 |
| TNFRSF12A | 0.36 |  | ANXA2P2 | 0.81 |  | AC073072.5 | 0.84 |
| RBM19 | 0.36 |  | RRAD | 0.81 |  | KCNE4 | 0.84 |
| TRIM71 | 0.36 |  | MMP14 | 0.81 |  | CPNE7 | 0.84 |
| DDX56 | 0.36 |  | RP11-359E10.1 | 0.81 |  | PDPN | 0.84 |
| MIF-AS1 | 0.35 |  | BDKRB1 | 0.81 |  | CTB-114C7.4 | 0.84 |
| HES6 | 0.35 |  | SMYD2 | 0.81 |  | RP5-944M2.3 | 0.84 |
| TBC1D22A | 0.35 |  | STPG1 | 0.8 |  | RP11-417E7.1 | 0.84 |
| AP001469.9 | 0.35 |  | DLGAP1 | 0.8 |  | KIAA1644 | 0.84 |
| FXYD6-FXYD2 | 0.35 |  | ZNF541 | 0.8 |  | CPNE5 | 0.84 |
| RP11-36I17.2 | 0.35 |  | C1orf198 | 0.8 |  | BICD1 | 0.83 |
| CELP | 0.35 |  | LINC00853 | 0.8 |  | CREB3L1 | 0.83 |
| ERGIC3 | 0.35 |  | DOK7 | 0.8 |  | TRPC4 | 0.83 |
| MRGBP | 0.35 |  | RHBDL2 | 0.8 |  | PCDH17 | 0.83 |
| AC091770.3 | 0.35 |  | TREM2 | 0.8 |  | SLC7A14 | 0.83 |
| RASSF7 | 0.35 |  | SEZ6L2 | 0.8 |  | MAP1B | 0.83 |
| GOLGA2P7 | 0.35 |  | TNFSF9 | 0.8 |  | MIR7-3HG | 0.83 |
| SAPCD1 | 0.35 |  | OLR1 | 0.8 |  | NANOGP7 | 0.83 |
| RP11-465B22.3 | 0.35 |  | SPINT1 | 0.8 |  | RP11-817I4.1 | 0.83 |
| RP11-529E10.6 | 0.35 |  | THAP2 | 0.8 |  | CDK17 | 0.83 |
| PDRG1 | 0.35 |  | NPPB | 0.8 |  | BARX2 | 0.83 |
| CDH9 | 0.35 |  | B3GNT3 | 0.8 |  | AP006285.2 | 0.83 |
| EIF3B | 0.34 |  | FGF13 | 0.8 |  | CCDC80 | 0.82 |
| KDELR1 | 0.34 |  | TGFBI | 0.79 |  | TMEM59L | 0.82 |
| PIGU | 0.34 |  | ESRRAP2 | 0.79 |  | N4BP3 | 0.82 |
| COX19 | 0.34 |  | HIST1H2AB | 0.79 |  | WBP5 | 0.82 |
| EIF6 | 0.34 |  | TMC7 | 0.79 |  | KCNF1 | 0.82 |
| CYP2W1 | 0.34 |  | CTD-2626G11.2 | 0.79 |  | RBM24 | 0.82 |
| PFDN6 | 0.34 |  | IGSF3 | 0.79 |  | ADAMTS12 | 0.82 |
| CLCN2 | 0.34 |  | MOXD1 | 0.79 |  | CDC42EP5 | 0.82 |
| MANBAL | 0.34 |  | STMN2 | 0.79 |  | CTHRC1 | 0.82 |
| DDX54 | 0.34 |  | PSG8 | 0.79 |  | FOXL1 | 0.82 |
| GET4 | 0.34 |  | SMKR1 | 0.79 |  | F2RL3 | 0.82 |
| TMEM120A | 0.34 |  | KRT23 | 0.79 |  | TPM4P1 | 0.82 |
| FBXW4P1 | 0.34 |  | CH17-224D4.2 | 0.79 |  | RP11-775D22.2 | 0.81 |
| ABHD12 | 0.34 |  | SPON2 | 0.78 |  | CRISPLD2 | 0.81 |
| ARFRP1 | 0.34 |  | CRCT1 | 0.78 |  | RP11-789C17.1 | 0.81 |
| LINC00336 | 0.34 |  | CXCL8 | 0.78 |  | SH3RF3 | 0.81 |
| ARVCF | 0.34 |  | LINC00473 | 0.78 |  | PGS1 | 0.81 |
| KRT18P27 | 0.34 |  | KRT80 | 0.78 |  | RP11-64B16.3 | 0.81 |
| SMUG1 | 0.34 |  | TMEM132A | 0.78 |  | RP11-411K7.1 | 0.81 |
| RAB24 | 0.34 |  | DAGLA | 0.78 |  | GIPR | 0.81 |
| GTF2IP1 | 0.34 |  | DEFB1 | 0.78 |  | AC007750.5 | 0.81 |
| GLUD2 | 0.34 |  | PSG9 | 0.78 |  | GFPT2 | 0.81 |
| TAF10 | 0.34 |  | ITGA3 | 0.78 |  | TMEM207 | 0.8 |
| NSUN5P2 | 0.34 |  | ARMC9 | 0.78 |  | RP11-675F6.4 | 0.8 |
| RP13-608F4.5 | 0.34 |  | DAXX | 0.78 |  | DNAJA4 | 0.8 |
| GUSBP11 | 0.34 |  | EDN2 | 0.78 |  | TNFRSF6B | 0.8 |
| C12orf49 | 0.34 |  | DCDC2 | 0.78 |  | SPHK1 | 0.8 |
| RP11-274B21.10 | 0.34 |  | COMP | 0.78 |  | HIVEP3 | 0.8 |
| TRNP1 | 0.34 |  | RP11-96A15.1 | 0.78 |  | MARCH3 | 0.8 |
| PLEKHB1 | 0.34 |  | GEM | 0.78 |  | CACNA1G | 0.8 |
| RP11-241F15.3 | 0.34 |  | CTHRC1 | 0.78 |  | SMARCD3 | 0.8 |
| SSUH2 | 0.34 |  | CCL2 | 0.78 |  | RP11-248J23.7 | 0.8 |

**Supplementary Figure legends**

**Figure S1** Protein-protein interaction analysis of AIM1L in STRING (A) and STITCH (B) databases.

**Figure S2** Differentially expressed related-genes of AIM1L screened by edgeR package in R program in TCGA dataset with a |log FC| > 1, and adjusted *P* value < 0.05.


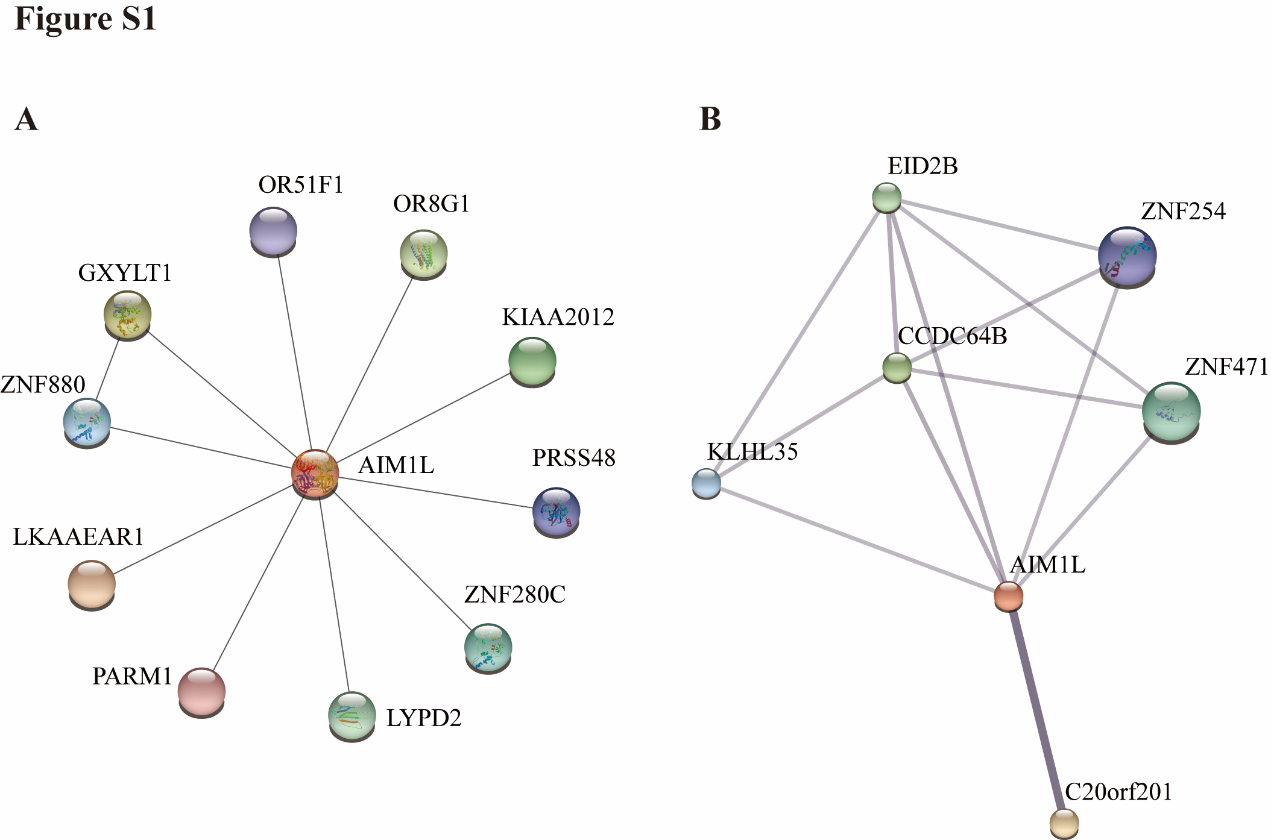


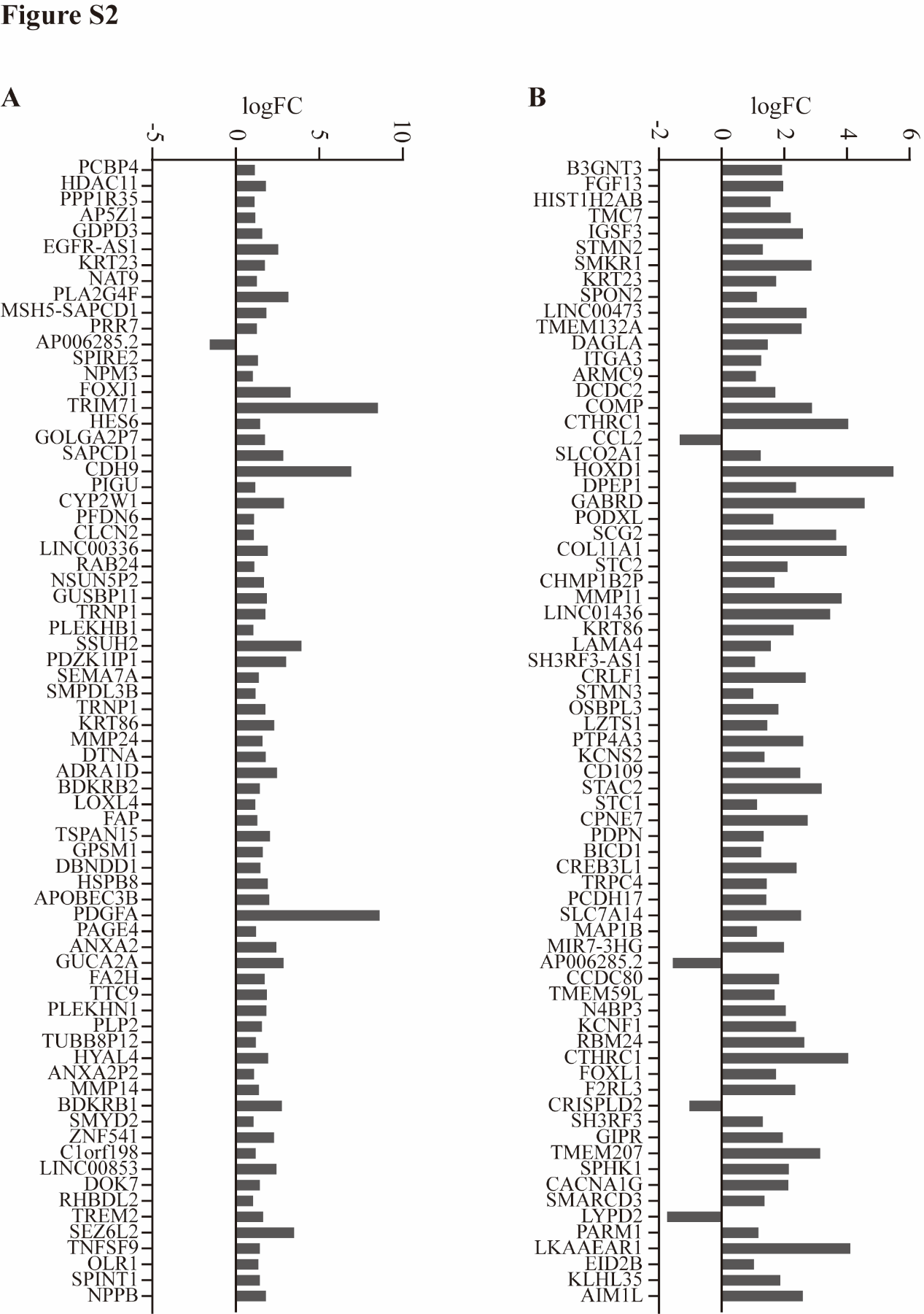

Supplement: Supplemental Material [file KBIE_A_1939636_SM4128.zip › supplementary/Supplementary Materials.docx]
